# Guillain–Barré Syndrome in Northern China: A Retrospective Analysis of 294 Patients from 2015 to 2020

**DOI:** 10.3390/jcm11216323

**Published:** 2022-10-26

**Authors:** Qiongqiong Zhai, Cheng Guo, Fang Xue, Jing Qiang, Chaonan Li, Li Guo

**Affiliations:** 1 Department of Neurology, The Second Hospital of Hebei Medical University, Shijiazhuang 050000, China; 2 Department of Pediatrics, The Third Hospital of Hebei Medical University, Shijiazhuang 050051, China; 3 Department of Pediatrics, The Second Hospital of Hebei Medical University, Shijiazhuang 050000, China

**Keywords:** Guillain–Barré syndrome, AMAN subtype, northern China, regional differences

## Abstract

Objectives: Acute motor axonal neuropathy (AMAN) was first reported to be the main subtype of Guillain–Barré syndrome (GBS) in northern China in the 1990s. About 30 years has passed, and it is unknown whether the disease spectrum has changed over time in northern China. We aimed to study the epidemiological, clinical, and electrophysiological features of GBS in northern China in recent years. Methods: We retrospectively analyzed the medical records of GBS patients admitted to the Second Hospital of Hebei Medical University in northern China from 2015 to 2020. Results: A total of 294 patients with GBS were enrolled, with median age 53 years and 60.5% of participants being male, and a high incidence in summer and autumn. AMAN was still the predominant subtype in northern China (40.1%). The AMAN patients had shorter time to nadir, longer hospitalization time, and a more severe HFGS score at discharge than acute inflammatory demyelinating polyneuropathies (AIDP) (*p* < 0.05). With SPSS multivariable logistic regression analysis, we found the GBS disability score (at admission), dysphagia, and dysautonomia were independent risk factors for GBS patients requiring MV (*p* < 0.05). In comparison with other regions, the proportion of AMAN in northern China (40.1%) was higher than in eastern (35%) and southern (19%) China. Conclusions: AMAN is still the predominant subtype in northern China after 30 years, but there have been changes over time in the GBS spectrum since the 1990s. There are regional differences in GBS in China.

## 1. Introduction

Guillain–Barré syndrome (GBS) is an acute onset, immune-mediated polyradiculoneuropathy and is a common cause of acute flaccid paralysis worldwide [1]. The reported incidence of GBS ranges from 0.81 to 1.89 cases per 100,000 person-years in north America and Europe [2]. In China, a recently published national incidence for GBS was 0.233 in children and 0.829 in adults per 100,000 [3]. GBS occurs more frequently in males than in females and the incidence increases with age, although all age groups can be affected. In most GBS patients, the acute onset of neurological symptoms is preceded by an infective illness. The typical clinical manifestation is progressive limb weakness or paresthesia, with decreased or absent tendon reflex and albumin–cytologic dissociations in cerebrospinal fluid (CSF). Clinical symptoms usually reach the nadir within 2 weeks. About 20% of GBS patients will develop respiratory failure and require mechanical ventilation [4]. Intravenous immunoglobulin (IVIg) and plasma exchange are proven to be effective treatment for GBS [5]. Acute inflammatory demyelinating polyneuropathies (AIDP) and acute motor axonal neuropathy (AMAN) are the two main subtypes of GBS. Less common subtypes of GBS include acute motor-sensory axonal neuropathy (AMSAN), Miller Fisher syndrome, pharyngeal–cervical–brachial, and bifacial weakness with paresthesia [6,7].

In the 1990s, there was an annual epidemic of GBS in summer in children and young adults from rural areas in the Hebei province of northern China. In cooperation with our hospital, Ho et al. [8] first discovered that AMAN was the main subtype of GBS in northern China, and confirmed the relationship between previous Campylobacter jejuni infection, anti-glycolipid antibodies and GBS [8,9], which had received worldwide attention. Since then, more research has been conducted in other regions in China. Researches showed that AIDP was the predominant form in southern [10], northeast [11], northwest China [12], and Hong Kong [13], while AMAN was the predominant form in eastern China [14]. Antecedent events, and clinical severity also differ in different regions. Differences in climate, diet, and medical level lead to obvious regional differences in China [10]. However, there is still a lack of research reports with large sample size in north China in recent years. The clinical data on GBS in northern China cited by international researchers are still the data obtained in the 1990s [7].

It has been more than 30 years since the 1990s, and whether the disease spectrum has changed over time in northern China is unknown. This study aims to study the epidemiological, clinical, and electrophysiological features of GBS patients in northern China from 2015 to 2020 in the Second Hospital of Hebei Medical University.

## 2. Materials and Methods

### 2.1. Patients

We retrospectively reviewed the medical records of GBS patients hospitalized in the Department of Neurology and Pediatrics of the Second Hospital of Hebei Medical University from 1 January 2015 to 31 December 2020. The patients were mainly from Hebei Province and surrounding provinces in northern China.

Inclusion criteria: (1) Patient diagnosed with GBS for the first time. (2) The Brighton criteria [15] for GBS were met: a natural history compatible with GBS, and the absence of an alternative diagnosis. The clinical features considered were monophasic course and 12 h to 28 days between onset and nadir, bilateral, flaccid weakness of limbs, diminished or absent deep tendon reflex, albumin–cytological dissociation of the cerebrospinal fluid, and nerve conduction study findings. (3) Patients whose clinical presentation and ancillary data were typical of GBS except for preservation or exaggeration of reflexes were also included. (4) Complete inpatient data.

Exclusion criteria: acute onset of chronic idiopathic polyneuropathy (A-CIDP), spinal cord disease, peripheral neuropathy due to other etiologies, and peripheral neuropathy with other diagnoses.

We extracted information regarding demographic data, antecedent event (infection, vaccination, other diseases), clinical symptoms (initial manifestations, muscle strength, sensory impairment, cranial nerve injury, autonomic dysfunction), auxiliary examination results (cerebrospinal fluid examination, clinical neurophysiological examination, and other related examinations), treatment, mechanical ventilation (MV), time to nadir, and hospital stay. We assessed the degree of disability of GBS (at admission, at nadir, and discharge) by using the Hughes Functional Grading Scale (HFGS) [16]. The time to nadir was defined as the point when the patient had the highest HFGS, or the time when the symptoms were no longer progressing. Autonomic dysfunction included instability of blood pressure (hypertension/hypotension), abnormal heart rate fluctuation, and sweating, and bowel and bladder incontinence or retention. Albumin–cytologic dissociation was defined as a combination of an increased protein level (>400 mg/dL) and normal cell count (<50 cells/μL) in CSF.

We also reviewed the previous GBS research data of northern China from the 1990s [8] and the data from recent studies in southern [17] and eastern [14] China for comparison.

### 2.2. Electrophysiological Studies

Electrophysiological studies were performed using standard procedures with a DANTEC key-point EMG machine (Dantec, Skovlunde, Denmark). In motor nerve conduction studies, at least one unilateral median, ulnar, and bilaterally peroneal nerve and F waves were recorded. In sensory nerve conduction studies, median, ulnar, and bilateral sural nerves were evaluated. A value was defined as abnormal if it fell outside our laboratory range of age-corrected normal responses.

Patients were classified as having demyelinating neuropathy (AIDP) and axonal neuropathy (AMAN) using the most widely used Hadden’s [18] electrophysiologic criteria. Patients whose nerve conduction studies showed absent motor evoked responses were classified as inexcitable and patients whose data did not conform to either category were considered equivocal. In addition, Ho and colleagues’ electrophysiological criteria [8], which were first proposed when studying GBS patients in our hospital in northern China were also used. A comparison of the two sets of electrophysiological criteria is shown in Supplementary Table S1.

### 2.3. Statistical Analysis

Continuous variables with skewed distribution were presented as median (Q1–Q3). The Kolmogorov–Smirnov test was used for the normality check. The categorical data were expressed as the number of cases or the constituent ratio (%). We used the Mann–Whitney U-test to compare continuous data with skewed distribution. Differences in proportions were tested with the χ^2^ or Fisher’s exact test. The “need for MV” were dichotomized into two dependent variables (presence/absence of need for MV = 0/1). Risk factors of need for MV in GBS patients were analyzed by multivariable logistic regression (step forward method).

A two-tailed *p* value < 0.05 meant the difference was statistically significant. Statistical analysis was performed with SPSS version 22.0 (IBM, Armonk, NY, USA) [19] and Graphpad-Prism version 8.00 (GraphPad Software, San Diego, CA, USA).

### 2.4. Ethical Approval

The study was approved by the Research Ethics Committee of the Second Hospital of Hebei Medical University (ethical code number: 2022-R572).

## 3. Results

### 3.1. General Features

**Figure 1:**
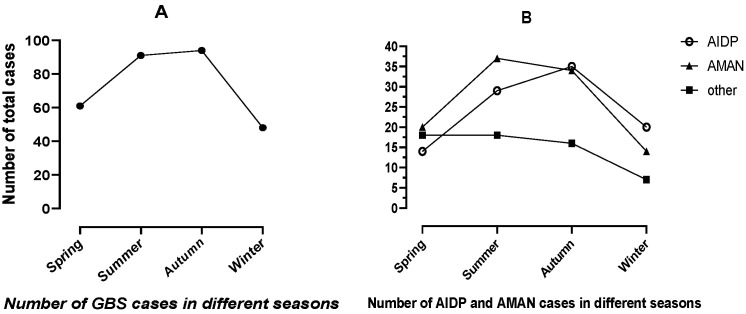
Number of GBS cases in different seasons. (**A**) Number of GBS cases in different seasons. (**B**) Number of GBS subtype cases in different seasons.

A total of 294 GBS patients, who were hospitalized in the Second Hospital of Hebei Medical University from 2015 to 2020, were included in this study. GBS patients showed seasonal changes, with a high incidence in summer and autumn (Figure 1A). AIDP was more common in autumn, AMAN was more common in summer, and other types of GBS patients were relatively more common in winter and spring (Figure 1B).

**Figure 2:**
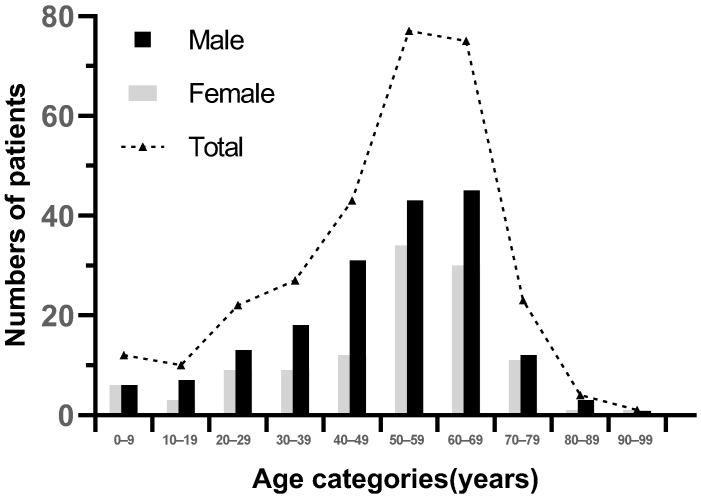
Age and gender distribution of GBS patients.

We summarized the clinical, laboratory, and electrophysiological subtypes of the 294 GBS patients in Table 1. The age was 53 (42.5–63) years. The number of patients increased with age, and peaked in the 50–69 year category (Figure 2). The male/female ratio was 1.53 (178 men and 116 women). Prior to developing neurological symptoms, 49% of patients with GBS had a previous infective event within 4 weeks. The initial symptoms were limb weakness in 189 cases (64.3%), paresthesia in 78 cases (26.5%), and cranial nerve symptoms in 27 cases (9.2%). The frequency of patients with absent or decreased tendon reflex was 87.1% (256/294) at entry. All patients reached peak within 4 weeks and the time to nadir was 6 (4–10) days. The clinical manifestations at nadir were mainly limb weakness, numbness, cranial nerve involvement and autonomic dysfunction. Cranial nerve involvement was mainly facial paralysis (59 cases), and 39 cases (13.3%) were mechanically ventilated for respiratory weakness. The length of hospital stay was 14 (10–20) days. Electromyography (EMG) was performed in 262 of these patients. The electrophysiological types were mainly AIDP (37.8%) and AMAN (40.1%) according to Hadden’s electrophysiologic criteria. CSF was examined in 221 patients and 85.1% patients (188/221) had albumin–cytological dissociation in the CSF.

### 3.2. Clinical Features of Different Subtypes (AIDP vs. AMAN)

We compared the characteristics of AIDP and AMAN patients (see Table 2). There was no significant difference between AIDP and AMAN subtypes in terms of age or gender. In the comparison of antecedent events, gastrointestinal infection was more common in AMAN patients, while upper respiratory tract infection (URTI) was more common in AIDP, but the difference did not attain statistical significance (*p* = 0.161). Additionally, patients with AIDP had a higher frequency of cranial nerve involvement (27.3% vs. 15.6% *p* = 0.054) and facial paralysis (21.2% vs. 8.5%, *p* = 0.011). Note that paresthesia was more frequent in AIDP patients than in AMAN patients (59.6% vs. 27.6%, *p* < 0.001). There was no significant difference in the HFGS score between the two groups at admission. However, the proportion of severe GBS patients at discharge (HFGS score ≥3) in the AMAN group was higher than that of the AIDP group (69.5% vs. 55.5%, χ² = 4.253, *p* = 0.039). The AMAN patients had shorter time to nadir, longer hospitalization time, and a more severe HFGS score at discharge than the AIDP group (*p* < 0.05).

### 3.3. Clinical Severity, Treatment and Outcome

Of all the patients enrolled, 218 patients (74.15%) presented with severe disease (Hughes disability grade ≥3, Table 1) at entry. 143 patients (48.6%) were treated with intravenous immunoglobulin (IVIg) and 117 (39.8%) patients were treated with both IVIg and glucocorticoids. The main reason for adding glucocorticoids was the poor clinical response to IVIg, mostly with a dosage of 5–10 mg dexamethasone or 40–80 mg methylprednisolone for 5 days or more. Moreover, 16 (5.4%) patients were with glucocorticoids alone and the remained 18 (6.2%) patients with only supportive treatment for mild symptoms or economic reasons (see Table 1). IVIG was expensive and not covered by medical insurance. IVIg treatment was started within 1–5 days after admission, and no obvious adverse reactions were found.

At discharge, 17 patients recovered, 105 patients (35.7%) presented with mild disease (GBS disability score 1 or 2), 61 patients (20.7%) were able to walk with a support (GBS disability score 3), and 97 patients (33%) were confined to bed or chair bound (GBS disability score 4); 10 patients (3.4%) required assisted ventilation (GBS disability score 5); 4 patients (1.4%) died due to severe complications. Elderly patients (≥60 years) had higher GBS disability scores compared with the other patients (<60 years) at discharge (*p* = 0.002). After discharge, most of the severe patients who could not walk unaided were transferred to rehabilitation institutions or received outpatient rehabilitation treatment for further improvement.

In our cohort, 39 patients (13.3%) required MV at nadir, and we compared the clinical characteristics of patients requiring MV with those not requiring MV (see Table 3). We found that patients requiring MV had higher HFGS scores at entry and higher CSF protein level, and a greater proportion of dysphagia and dysautonomia (*p* < 0.05). With SPSS multivariable logistic regression analysis, we found that the GBS disability score (at admission), dysphagia, and dysautonomia were independent risk factors for GBS patients requiring MV (all *p* < 0.05). Table 4 shows the multivariable logistic regression analysis.

In order to be consistent with Ho’s previous study [8] in our hospital in 1995 and to rule out the influence of different electrophysiological criteria, we reclassified the 262 patients with EMG in our cohort according to Ho’s electrophysiological criteria (Table 5). We found 15 patients with AIDP and 2 patients considered equivocal were reclassified as AMAN, 7 patients with AIDP were reclassified as equivocal according to Ho’s criteria and the proportion of AMAN was 46.6% followed by AIDP (29.4%). AMAN was the predominant subtype under both sets of electrophysiological criteria.

### 3.4. Regional Comparison of GBS Patients in China

We conducted a horizontal comparison of the relatively large-scale clinical and epidemiological retrospective analysis of GBS in China (southern China [17], eastern China [14]) published in recent years (Table 6). There was a statistical difference in the proportion of the electrophysiological subtype and pro-infection between the three regions (*p* < 0.001). AMAN was the predominant subtype in northern (40.1%) and eastern China (35%), while AIDP was predominant in southern China (49%). Additionally, the proportion of AMAN in northern China was higher than in eastern (35%) and southern (19%) China. Gastrointestinal infection was the main infection trigger in the north (23.5%), while upper respiratory tract infection was the main infection trigger in the south (35%) and east (29%). Cranial nerve damage was more common in GBS patients in eastern and southern China (*p* < 0.01), while MV was more common in northern and eastern China (*p* = 0.004). In term of sensory deficits, there was no difference among the three areas.

## 4. Discussion

### 4.1. Epidemiology Feature

Overall, 294 patients with GBS were identified in our hospital from 2015–2020. We found that there was a higher proportion of male than female, which was consistent with other studies [20,21]. The incidence of GBS in both men and women increased with age, peaking at the age 50–69 years. A similar conclusion was found in other regions worldwide [2]. However, in the early 1990s, GBS patients were mainly children and young adults, which indicated that the onset age of GBS has changed [22].

There were regional differences in the seasonality of GBS [23]. The incidence of GBS patients was higher in winter in Australia [20], Italy [22], and spring in Greece [23]. In our study, an increased incidence of GBS was observed in summer and autumn, which was consistent with the report in the 1990s [9] and the report by Islam et al. [24]. The distribution of GBS cases in the seasons may be related to the climatic zone and infection in different regions.

### 4.2. Antecedent Events

About 64.3% of the patients had antecedent events within 4 weeks before symptomatic onset. In accordance with other studies [6], the most frequent prodromic events were upper respiratory tract infection and gastroenteritis. Guillain–Barré syndrome has also been associated with particular vaccinations [25], tumor [26], and autoimmune disease. Other triggers such as trauma and surgery were rarely reported [27].

Regarding the pre-infection events, there were regional differences in China. The upper respiratory tract infections were the main trigger in southern and eastern China, while the gastrointestinal infections were the main trigger in northern China, which may be related to geographical differences in environmental characteristics and infection [17]. Disease spectrum has changed over time in northern China. The outbreak of Campylobacter jejuni infection in the summer of 1990s in Hebei Province led to a sharp increase in GBS in children and young adults [8]. In recent years, the outbreak of Campylobacter jejuni infection was not common, because of the improvement in environmental and dietary hygiene conditions. In the late 20th century, people in the rural area in north China usually drunk unboiled water in the river with animal wastes in it; poultry was also free-range, and there were no hygiene habits of hand-washing before meals. The proportion of gastrointestinal and respiratory infections in antecedent event decreased significantly in the present study compared to the previous study in the 1990s. These changes may also be related to the changes in the disease spectrum over time.

The role of vaccination as a possible trigger of GBS is controversial. Previous research had shown an increased risk of Guillain–Barré syndrome in individuals receiving the 1976 H1N1 influenza vaccine [28], other subsequent studies had shown that influenza vaccination/other vaccinations did not increase the incidence of GBS [29,30,31]. In our study, only two patients developed GBS within a few weeks after the influenza vaccination. Currently, there is a lack of evidence to support that vaccination against COVID-19 increases the incidence of GBS [32]. Most of the Chinese population have been vaccinated against COVID-19 since 2020, and no related GBS patients have been reported. Overall, vaccination does not increase the incidence of GBS in north China, but it still needs to be further confirmed by large-scale prospective studies.

Cancer may increase the risk of GBS due to molecular mimicry or immunosuppression, but the exact relationship is unclear. Levison et al. [26] reported that 2% of GBS patients in Denmark had a recent cancer diagnosis, between 6 months before and 2 months after the GBS, with the highest incidences of respiratory cancer, breast cancer, prostate cancer, and lymphocyte cancer. Our study had five (1.7%) cancers (four breast cancers and one lung cancer), diagnosed 6 to 2 months before GBS, with a history of surgery or chemotherapy. The clear and common immunopathogenesis linking cancer and GBS development is not well understood, and it may be associated with some unrecognized factors present in cancer.

Trauma or surgery is a rare trigger for GBS, and there are increasingly more clinical reports in recent years [27]. Our study had 32 patients (10.9%) with a history of surgery or trauma before GBS, mainly craniocerebral trauma (14 cases), craniocerebral surgery (4 cases), spine surgery (6 cases), and 8 cases of surgery on other parts (extremities, heart, abdomen, thyroid, lung), consistent with the meta-analysis by Huang et al. [27]. Trauma or surgery may produce abnormal immune and inflammatory responses that increase the occurrence of GBS, by causing psychological stress, physical damage, and blood–brain barrier disruption.

### 4.3. Clinical Features of Different Electrophysiological Subtypes and Regional Variation

Previous epidemiological studies showed that the predominant electrophysiological subtype was demyelinating in some regions (Europe/Americas: *n* = 312/573, 55%; Asia: *n* = 29/65, 45%; Bangladesh: *n* = 38/94, 40%) [33]. Our study found that AMAN remained to be the predominant electrophysiological subtype in northern China, which was consistent with the results of the 1990s [9] and eastern China studies [14]. Regional differences in GBS clinical and electrophysiological subtype frequencies may be partly due to differences in local infection exposure. With the improvement in sanitary conditions, the incidence of diarrhea caused by Campylobacter jejuni infection has decreased significantly, but AMAN was still the main type, which may be related to environmental characteristics, geographical differences in genetic polymorphisms of Campylobacter jejuni strains, and genetic polymorphisms between populations living in different regions [34,35]. In addition, we found that AMAN progressed more rapidly and had a worse prognosis than AIDP. Studies in southern China [17] and other countries [33] also showed the similar result that AMAN were associated with relatively poor recovery.

GBS as a peripheral neuropathy, is regarded to be characterized by decreased tendon reflexes, and normal or hyperactive deep tendon reflexes are rare. However, previous studies [36,37,38] showed that 5–12% of patients had normal or hyperactive tendon reflexes. In our study, we found that tendon reflexes were normal in 13 (13.1%) of AIDP, 12 (11.4%) of AMAN, and 4 (3.8%) of AMAN had hyperreflexia on admission. All these people later developed diminished or absent tendon reflexes. The mechanism of hyperreflexia in GBS patients is unclear. However, this finding suggests that GBS cannot be ruled out if tendon reflexes are preserved or hyperactive in the early stage of the disease, which is also a supplement to the traditional GBS diagnostic criteria.

### 4.4. Clinical Features of Mechanically Ventilated Patients

Respiratory muscle involvement is the most dangerous complication of GBS. Approximately 40% of children with GBS develop respiratory muscle weakness requiring MV [39], while approximately 20–30% of adults with GBS require MV [40]. In our cohort, 39 patients (13.3%) required MV, which is significantly lower than in previous studies (39%) [7]. We compared the clinical characteristics of patients requiring MV with those not requiring MV and identified higher GBS disability scores (at entry, at nadir) and CSF protein, dysphagia, and dysautonomia as factors significantly associated with MV. Multivariable logistic regression analysis showed that higher HFGS score on admission, dysphagia, and dysautonomia were risk factors for GBS requiring MV, but mechanical ventilation was not associated with GBS subtype, age, sex, and pre-infection, which was consistent with descriptions in other literature [41,42]. Dysphagia is a bulbar paralysis syndrome related to the glossopharyngeal and vagal nerve deficits in GBS and dysphagia was reported to be a risk factor for MV in the literature. Expiratory weakness and impairment of coughing combined with bulbar dysfunction may lead to aspiration, accumulation of airway secretions, and worsening of respiratory failure [43,44].

The incidence of MV in our study was 13.3%, which was lower than other literature values (20–30% of adults with GBS) [40], the possible reason may lie in the early administration of intravenous immunoglobulin (1–5 days after admission, 260/294, 88.43%) and glucocorticoid therapy to block disease progression. Early identification of GBS patients at risk of developing MV can improve the prognosis of GBS patients, and models for predicting the risk of developing MV in GBS patients after admission are clinically meaningful and beneficial.

### 4.5. Limitations

There are still some deficiencies in our research. First, the retrospective nature of the study could cause selection bias in the study. Second, our study lacked long-term follow-up work of the patients enrolled, so it was not possible to analyze the long-term prognosis of the GBS patients. Third, because the cost of GBS-related antibody testing is relatively expensive and not covered by medical insurance, most patients refuse related testing, and there is a lack of ganglioside antibody analysis.

## 5. Conclusions

GBS has obvious regional differences, the epidemiological and clinical characteristics of GBS in different countries or different regions in China are significantly different. This study updated the clinical characteristics and epidemiological information of GBS in Hebei Province, northern China. The subtypes of GBS in northern China are still dominated by the axonal subtype, patients were mainly 50–69 years old, and gastrointestinal and respiratory tract infections are still the major prodromal event. Early identification of GBS patients at risk for MV can reduce adverse outcomes associated with delayed intubation. Preserved or hyperactive tendon reflexes in the early stage of the disease cannot rule out GBS, suggesting that the diagnostic criteria for GBS still need to be continuously improved. Future work should focus on models for predicting clinical course and severity to improve the prognosis of GBS.

## Figures and Tables

**Table 1:** Epidemiological, clinical, laboratory, and electrophysiological features of GBS (*n* = 294).

Parameters	
Age * [years, M (Q1–Q3)]	53 (42.5–63)
**Male, *n* (%)**	178 (60.5%)
**Antecedent events within 4 weeks**	
URTI, *n* (%)	64 (21.8%)
Gastroenteritis, *n* (%)	69 (23.5%)
Pneumoniae, *n* (%)	8 (2.7%)
Vaccination, *n* (%)	2 (0.6%)
Varicella-zoster virus, *n* (%)	3 (1%)
Cancer, *n* (%)	5 (1.7%)
Autoimmune diseases, *n* (%)	7 (2.4%)
Surgery or trauma, *n* (%)	32 (10.9%)
Others or none, *n* (%)	104 (35.4%)
**Electrophysiological subtype**	
AIDP, *n* (%)	99/262 (37.8%)
AMAN, *n* (%)	105/262 (40.1%)
Equivocal, *n* (%)	28/262 (10.7%)
Inexcitable, *n* (%)	6/262 (2.3%)
Normal, *n* (%)	24/262 (9.1%)
**Initial symptoms**	
Motor weakness, *n* (%)	189 (64.3%)
Sensory change, *n* (%)	78 (26.5%)
Cranial nerve palsy, *n* (%)	27 (9.2%)
**Tendon reflex at entry**	
Hyperreflexia, *n* (%)	4 (1.4%)
Absent or decreased tendon reflex, *n* (%)	256 (87.1%)
Normal tendon reflex, *n* (%)	34 (11.5%)
**Clinical Presentation at nadir**	
Limb weakness, *n* (%)	271 (92.2%)
Paresthesia, *n* (%)	131 (44.6%)
Facial paralysis, *n* (%)	59 (20.1%)
Bulbar palsy, *n* (%)	38 (12.9%)
Diplopia, *n* (%)	37 (12.6%)
Autonomic dysfunction, *n* (%)	51 (17.3%)
Mechanical ventilation (MV), *n* (%)	39 (13.3%)
**HFGS score**	
HFGS score at entry ≥3, *n* (%)	218 (74.1%)
HFGS score at nadir ≥3, *n* (%)	232 (78.9%)
HFGS score at discharge ≥3, *n* (%)	172 (58.5%)
Time to nadir * [d,M (Q1–Q3)]	6 (4–10)
Hospital stay * [d,M (Q1–Q3)]	14 (10–20)
CSF protein concentration * [g/L,M (Q1–Q3)]	0.8 (0.5–1.36)
**CSF albumin-cytologic dissociations, *n* (%)**	188 (85.1%)
**Treatment, *n* (%)**	
IVIg treatment alone, *n* (%)	143 (48.6%)
IVIg plus steroids, *n* (%)	117 (39.8%)
Steroids, *n* (%)	16 (5.4%)
Supportive treatment, *n* (%)	18 (6.2%)

URTI, upper respiratory tract infection; AIDP, acute inflammatory demyelinating polyneuropathies; AMAN, acute motor axonal neuropathy; IVIg, intravenous immunoglobulin; HFGS, the Hughes Functional Grading Scale; *n*, number; *, continuous variables with skewed distribution.

**Table 2:** Clinical features of different subtypes (AIDP vs. AMAN).

	AIDP (*n* = 99)	AMAN (*n* = 105)	Two-Tailed *p*-Value
Age * [years, M(Q1–Q3)]	53 (41–63)	53 (45–63)	0.882
**Male, *n* (%)**	63 (63.6%)	64 (61%)	0.693
**Antecedent events within 4 weeks**			0.161
URTI, *n* (%)	25 (25.3%)	18 (17.1%)	0.156
Gastroenteritis, *n* (%)	20 (20.2%)	31 (29.5%)	0.124
**Initial symptoms, *n* (%)**			**<0.001**
Motor weakness, *n* (%)	58 (58.6%)	91 (86.7%)	**<0.001**
Sensory change, *n* (%)	36 (36.4%)	12 (11.4%)	**<0.001**
Cranial nerve palsy, *n* (%)	5 (5.1%)	2 (1.9%)	0.268
**Clinical Presentation at nadir**			
Cranial nerve involvement, *n* (%)	27 (27.3%)	17 (15.6%)	0.054
Paresthesia, *n* (%)	59 (59.6%)	29 (27.6%)	**<0.00** **1**
Facial paralysis, *n* (%)	21 (21.2%)	9 (8.5%)	**0.011**
Autonomic dysfunction, *n* (%)	15 (15.2%)	16 (15.2%)	0.986
Mechanical ventilation (MV), *n* (%)	8 (8.1%)	6 (5.7%)	0.504
HFGS score at admission *,M (Q1–Q3)	3 (3–4)	4 (3–4)	0.120
HFGS score at discharge * [M (Q1–Q3),mean rank]	3 (2–4), 91.56	3 (2–4), 112.82	**0.007**
**HFGS score at discharge ≥3, *n* (%)**	55 (55.5%)	73 (69.5%)	**0.039**
Time to nadir * [d,M (Q1–Q3)]	7 (5–10)	6 (4–8.5)	**0.012**
Hospital stay * [d,M (Q1–Q3)]	13 (10–17)	15 (11–21.5)	**0.016**
CSF protein concentration * [g/L,M (Q1–Q3)]	0.97 (0.5–1.91)	0.8 (0.52–1.3)	0.473

URTI, upper respiratory tract infection; AIDP, acute inflammatory demyelinating polyneuropathies; AMAN, acute motor axonal neuropathy; HFGS, the Hughes Functional Grading Scale; *n*, number; *, continuous variables with skewed distribution and compared with the Mann–Whitney U-test.

**Table 3:** Comparison between patients requiring mechanical ventilation (MV) and the other GBS patients.

	Patient Requiring MV	Other Patients	*p*-Value
	**39 (13.3%)**	**255 (86.7%)**	
Age * [years, M (Q1–Q3)]	56 (40–63)	53 (42–62)	0.330
Gender Female/Male	17/22	99/156	0.571
Time from onset to admission * [d,M (Q1–Q3)]	7 (7–10)	9.5 (7–14)	0.204
HFGS score at admission *, M (Q1–Q3)	4 (4–4)	3 (2–4)	**<0.001**
HFGS score at nadir *, M (Q1–Q3)	5 (5–5)	4 (3–4)	<0.001
Electrophysiological subtype, *n*	AIDP 8, AMAN 6	AIDP90, AMAN99	N/A
Cranial nerve involvement, *n* (%)	15 (38.4%)	77 (30.2%)	0.300
Pre-infection, *n* (%)	22 (56.4%)	127 (49.8%)	0.442
CSF protein concentration * [g/L,M (Q1–Q3)]	1.08 (0.78–3.1)	0.78 (0.5–1.33)	**0.015**
Dysphagia, *n* (%)	12 (30.7%)	26 (10.2%)	**0.001**
Motor weakness, *n* (%)	22 (56.4%)	167 (65.5%)	0.270
Dysautonomia, *n* (%)	14 (35.8%)	37 (14.5%)	**0.002**

AIDP, acute inflammatory demyelinating polyneuropathies; AMAN, acute motor axonal neuropathy; HFGS, the Hughes Functional Grading Scale; *n*, number; *, continuous variables with skewed distribution and compared with the Mann–Whitney U-test; N/A, Not Available.

**Table 4:** Multinomial logistic regression model for variables associated with MV.

Variables	β	OR	95% Cl		*p*-Value
GBS disability score(at admission)	1.693	5.437	1.864	15.857	**0.002**
Dysphagia	1.472	4.360	1.009	18.835	**0.049**
Dysautonomia	1.874	6.513	1.689	25.120	**0.007**
CSF protein concentration	0.230	1.259	0.743	2.133	**0.391**

The GBS disability score was dichotomized according to the following criterion: GBS score 1 or 2: 0; GBS 3-5:1. CI, confident interval; GBS, Guillain–Barrè Syndrome; OR, odds ratio.

**Table 5:** Electrophysiological subtypes under different diagnostic criteria of GBS patients (*n* = 262).

	Hadden’s Criteria	Ho’s Criteria
AMAN, *n* (%)	105 (40.1)	122 (46.6)
AIDP, *n* %	99 (37.8)	77 (29.4)
Equivocal, *n* (%)	28 (10.7)	33 (12.6)
Inexcitable, *n* (%)	6 (2.3)	6 (2.3)
Normal, *n* (%)	24 (9.1)	24 (9.1)

AIDP, acute inflammatory demyelinating polyneuropathies; AMAN, acute motor axonal neuropathy.

**Table 6:** Regional Comparison of GBS patients in China.

Characteristic	Northern China *n* = 294	Eastern China *n* = 595	Southern China *n* = 1056	*p*-Value *	*p*-Value #
**Antecedent infection, *n* (%)**	144 (49)	271 (46)	458 (43)		
URTI, *n* (%)	64 (21.8)	175 (29)	369 (35)	0.021	8.8 × 10^−5^
Gastroenteritis, *n* (%)	69 (23.5)	96 (16)	89 (8)	0.012	5.8 × 10^−11^
**Cranial nerve involvement** **, *n* (%)**	92 (31.3)	256 (43)	435 (41)	0.003	0.004
**Sensory deficits** **, *n* (%)**	131 (44.6)	268 (45)	506 (48)	0.891	0.370
**Mechanical ventilation, *n* (%)**	39 (13.3)	68 (11)	83 (8)	0.467	0.007
**Electrophysiology classification**					
AIDP, *n* (%)	99/262 (37.8)	98/430 (23)	324/661 (49)	8.8 × 10^−5^	0.004
AMAN, *n* (%)	105/262 (40.1)	151/430 (35)	124/661 (19)		
Equivocal, *n* (%)	28/262 (10.7)	109/430 (25)	207/661 (31)		
Inexcitable, *n* (%)	6/262 (2.3)	7/430 (2)	6/661 (1)		
Normal, *n* (%)	24/262 (9.1)	65/430 (15)			

*: adjust *p* value, northern vs. eastern; #: adjust *p* value, northern vs. southern; URTI, upper respiratory tract infection; AIDP, acute inflammatory demyelinating polyneuropathies; AMAN, acute motor axonal neuropathy; *n*, number.

## Data Availability

The data presented in this study are available on request from the corresponding author. The data are not publicly available due to privacy.

## Supplementary Materials

The following supporting information can be downloaded at: https://www.mdpi.com/article/10.3390/jcm11216323/s1, Table S1: Comparison of Ho’s electrophysiological criteria and Hadden’s electrophysiological criteria.
