# Supplementary material for: Guillain–Barré Syndrome in Northern China: A Retrospective Analysis of 294 Patients from 2015 to 2020
Source: J Clin Med. 2022 Oct 26;11(21):6323. doi: 10.3390/jcm11216323 (PMC9658830; doi:10.3390/jcm11216323)
Supplement: Supplementary file 1 [file jcm-11-06323-s001.zip › jcm-1910942-supplementary.pdf]

Table S1. Comparison of Ho's electrophysiological criteria and Hadden's electrophysiological criteria

**Parameters**	**Ho criteria**	**Hadden criteria**
**AIDP** :At least one of the following in each of at least two nerves, or at least two of the following in one nerve if all others inexcitable and dCMAP>10% LLN		
Motor Conduction velocity (m/s)	<90% LLN (85% if dCMAP <50% LLN)	<90% LLN (85% if dCMAP <50% LLN)
Distal Latency (ms)	>110% ULN (>120% if dCMAP <100% LLN)	>110% ULN (>120% if dCMAP <100% LLN)
F-wave latency (ms)	>120% ULN	>120% ULN
Temporal dispersion	Evidence of unequivocal temporal dispersion	Not considered
Conduction block	Not considered	pCMAP/dCMAP ratio <0.5 and dCMAP>20% LLN
**AMAN**	①None of the features of AIDP except one demyelinating feature allowed in one nerve if dCMAP<10% LLN ②dCMAP <80% LLN in at least two nerves	
**Inexcitable**	dCMAP absent in all nerves or present in only one nerve with dCMAP <10% LLN	
**Equivocal**	Not conform to either category above	

dCMAP=compound muscle action potential amplitude after distal stimulation; pCMAP=compound muscle action potential amplitude after proximal stimulation; LLN=lower limit of normal. ULN=upper limit of normal.
